# SRT1720 promotes survival of aged human mesenchymal stem cells via FAIM: a pharmacological strategy to improve stem cell-based therapy for rat myocardial infarction

**DOI:** 10.1038/cddis.2017.107

**Published:** 2017-04-06

**Authors:** Xianbao Liu, Dexing Hu, Zhiru Zeng, Wei Zhu, Na Zhang, Hong Yu, Han Chen, Kan Wang, Yingchao Wang, Lengmei Wang, Jing Zhao, Ling Zhang, Rongrong Wu, Xinyang Hu, Jian'an Wang

**Affiliations:** 1 Department of Cardiology, Second Affiliated Hospital, Zhejiang University School of Medicine, Hangzhou, Zhejiang, China; 2 Provincial Key Laboratory of Cardiovascular Research, Hangzhou, Zhejiang, China; 3 Department of Cardiology, Ningbo Medical Center Lihuili Eastern Hospital, Ningbo, China

## Abstract

SIRT1 has been proved to rejuvenate and improve the therapeutic efficacy of aged rat mesenchymal stem cells (MSCs). Herein, we investigate the protective effect of pretreatment with SIRT1 activator SRT1720 on aged human MSCs (hMSCs). The optimized pretreatment condition for aged hMSCs was determined to be 0.5 *μ*M SRT1720 for 24 h by monitoring the survival of aged hMSCs subjected to serum deprivation±hypoxia and±500 *μ*M hydrogen peroxide (H_2_O_2_). Pretreatment with these conditions increased the survival of aged hMSCs 1 day (2.7-fold) and 3 days (1.9-fold) after being transplanted into a rat myocardial infarction (MI) model created by ligation of the left anterior descending (LAD) coronary artery. Transplantation with SRT1720 pretreated aged hMSCs achieved increased left ventricular ejection fraction (58.9±3.6 *versus* 52.8±5%) and angiogenesis with reduced fibrosis of rat hearts as compared to DMSO pretreated group 28 days following MI. Unbiased transcriptome analysis conducted on aged hMSCs under oxidative stress indicated the Fas apoptosis inhibitory molecule (FAIM) was significantly upregulated following SRT1720 pretreatment (14.9±0.2-folds). Moreover, the anti-apoptotic effect of SRT1720 was mitigated by FAIM knockdown with a small interfering RNA-targeted FAIM. These results indicated that pretreatment with SRT1720 improves survival of aged hMSCs, and enhances their therapeutic efficacy for rat myocardial infarction (MI). Upregulation of FAIM possibly involves in the mechanisms of the protective effects.

Transplantation of MSCs has been shown to be safe and is a promising strategy for the treatment of heart diseases.^1, 2, 3, 4^ However, survival and engraftment of MSCs following transplantation remains low, thus representing a major barrier for the overall therapeutic efficacy and utility of this approach. Moreover, it has been reported that MSCs from aged donors display a reduced ability to repair the heart in animal models.^5, 6^ The biological properties of human MSCs (hMSCs) including proliferation, differentiation potential and stress resistance decline with age and may limit the applications of these cells for clinical therapy.^7, 8, 9^ Thus, it's a big challenge to overcome the age-related dysfunction of hMSCs.

Silent information regulator 2 homolog 1 (SIRT1), also known as sirtuin 1, is a NAD^+^ dependent histone deacetylase that has important roles in metabolism and age-related pathologies including type 2 diabetes and neurodegenerative diseases.^10, 11^ SIRT1 has also been shown to positively regulate cell survival and apoptosis, as well as the responses to stress and inflammation through non-histone targets such as p53, FOXOs and NF-kB.^12, 13, 14^ In our previous study, we demonstrated that overexpression of SIRT1 conferred rejuvenation of aged rat MSCs and supported improved therapy in a rat MI model.^15, 16^ The findings support a practical strategy to rejuvenate and improve cell therapy by aged hMSCs via augmentation of SIRT1.

As the natural compound resveratrol was identified as a SIRT1 activator, a large number of small molecules have been found to activate SIRT1 (refs 17, 18 of which SRT1720 is the most effective and specific one.^19^ SRT1720 treatment extends the lifespan of both healthy mice and those on high fat diets,^20, 21^ and can ameliorate the disturbed flow induced senescence of endothelial cells.^22^ However, little is known on the effects of SRT1720 on normal human cells including human MSCs.

In the present study, we determined that SIRT1 expression is downregulated in aged hMSCs and this correlates with an impaired ability to resist stress. We further demonstrated that pretreatment of aged hMSCs with SRT1720 conferred improved cell survival and enhanced therapy in a rat MI model. Our results support a role for the Fas apoptosis inhibitory molecule (FAIM), in mediating the positive survival and pro-therapeutic actions of SRT1720 pretreatment by aged hMSCs.

## Results

### Characterization of aged hMSCs

**Figure 1:**
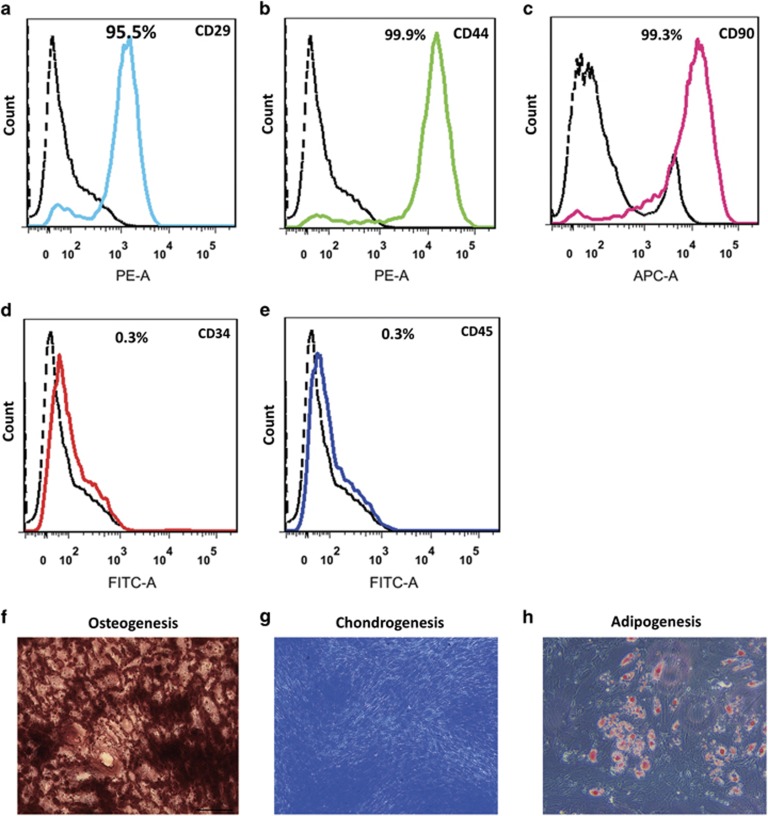
Characterization of hMSCs. Nearly all of the cells acquired expressed the cell surface markers PE-CD29 (**a**), PE-CD44 (**b**), APC-CD90 (**c**), and negative for the endothelial cell surface marker FITC-CD34 (**d**) and hematopoietic surface marker FITC-CD45 (**e**). The osteogenesis, chondrogenesis and adipogenesis differentiation of hMSCs was induced and visualized by alizarin red staining (**f**, dark red), toluidine blue staining (**g**, dark blue), and oil red O staining (**h**, red), respectively

Cell surface markers of the hMSCs were determined by flow cytometry. Nearly all of the cells acquired in our study were positive for the mesenchymal stem cell (MSC) surface markers: CD29, CD44, CD90 and negative for the endothelial cell surface marker CD34 and hematopoietic surface marker CD45, indicating characteristics of MSCs (Figures 1a–e). In addition, the multi-lineage differentiation capacity of hMSCs has also been tested, and they were proved to be able to differentiate into osteocytes, chondrocytes, and adipocytes (Figures 1f–h).

### Comparison of young and aged hMSCs reveals an association of SIRT1 with declined performances of aged hMSCs

In our previous studies, we have demonstrated that aged rat MSCs performed more expression of the cell senescence marker, *β*-galactosidase and weaker resistance to peroxide stress coupling with the low expression and activity of SIRT1 compared with young rat MSCs.^15, 16^ Similarly, expression of *β*-galactosidase was increased in aged hMSCs compared with young cells (OMSC 10.2±4.3 *versus* YMSC 2.7±1%, *P*=0.03) (Supplementary Figures 1a and b). To investigate a possible relationship between SIRT1 and cell viability of young *versus* aged hMSCs, we evaluated cell survival under conditions of imposed oxidative stress induced by serum deprivation combined with 500 *μ*M hydrogen peroxide (H_2_O_2_). Resistance to stress in aged hMSCs was significantly decreased compared to young hMSCs as indicated by survival indices (OMSC 44.6±4.7 *versus* YMSC 61.2±3.6%, *P*=0.03) (Supplementary Figures 1c). Aged hMSCs also displayed decreased expression and activity of SIRT1 (Supplementary Figures 1d and e). These results indicate a correlation between SIRT1 expression and resistance to stress in aged hMSCs.

### SRT1720 improves the survival of aged hMSCs *in vitro* and *in vivo*

**Figure 2:**
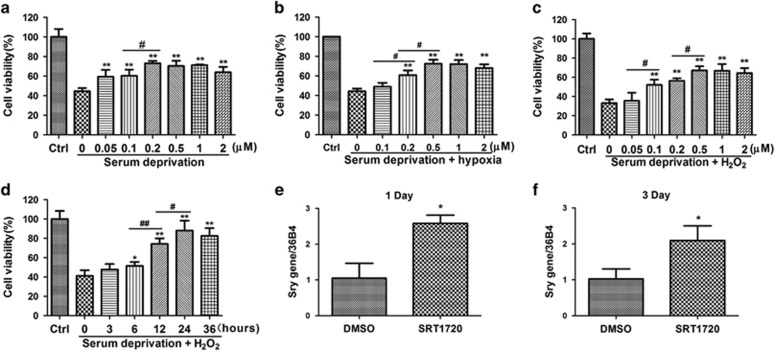
SRT1720 pretreatment promotes the survival of aged hMSCs under stress *in vitro* and *in vivo*. Protective effect of various concentration (0, 0.05, 0.1, 0.2, 0.5, 1, 2 *μ*M) of SRT1720 was evaluated under the situations of serum deprivation (**a**), serum deprivation and hypoxia (**b**), serum deprivation and H_2_O_2_ (**c**) by CCK-8 analysis. The survivals of aged hMSCs pretreated with 0.5 *μ*M SRT1720 for different time (0, 3, 6, 12, 24, 36 h) are detected under serum deprivation combined H_2_O_2_ situation (**d**). *SRY* gene expression of hMSCs in the whole heart was analyzed at day 1 (**e**) and day 3 (**f**) after cell therapy. The control condition (Ctrl) was normal culture medium (DMEM with 10% FBS). The volume fraction of DMSO was the same in every groups. Data are expressed by mean±S.D. (three independent experiments, *N*=3). *P<0.05 *versus* DMSO group; **P<0.01 *versus* DMSO group; ^#^*P*<0.05

To investigate whether decreased activity of SIRT1 was responsible for the reduced stress resistance of aged hMSCs, we used a specific activator of SIRT1, SRT1720, to stimulate SIRT1 activity in these cells. To mimic the low nutrition and hypoxic environment in the ischemic heart, three *in vitro* stress models were established and used to evaluate cell survival. The models include: serum deprivation, serum deprivation under hypoxia, and serum deprivation combined with H_2_O_2_. Respectively, cells were exposed to 24 h of serum deprivation or serum deprivation plus hypoxia, a time where ~50% cells survived the latter condition compared to normal culture without stress (Supplementary Figures 2a and b). In the setting of serum deprivation combined with H_2_O_2_, a concentration of 500 *μ*M H_2_O_2_ for 3 h was selected to induce ~50% cell survival compared to normal culture without stress (Supplementary Figure 2c). In these models, under the serum deprivation condition, SRT1720 pretreatment exhibited a dose-dependent protective effect on aged hMSCs, with the highest survival rate reached at a concentration of 0.2 *μ*M SRT1720 (73.0±2.5%) with no further benefits observed at higher concentrations (Figure 2a). However, when aged hMSCs were exposed to serum free medium combined with either hypoxia or 500 *μ*M H_2_O_2_, a concentration of 0.5 *μ*M was needed for maximal survival benefit (72.4±7.5% under hypoxia, 67.0±4.2% under H_2_O_2_ stress) (Figures 2b and c). Therefore, a concentration of 0.5 *μ*M was determined to be the optimized concentration of SRT1720 treatment for aged hMSCs. We further found that 24 h of pretreatment with 0.5 *μ*M SRT1720 was required to achieve maximum survival of aged hMSCs cultured in serum deprivation combined with 500 *μ*M H_2_O_2_ (88±9.6% Figure 2d).

To determine whether enhanced survival *in vitro* translated into improved survival *in vivo*, SRT1720 or vehicle pretreated aged hMSCs were injected into infarcted rat hearts and the male sex determination gene *sry* copy number within the recipient hearts was measured at 1, 3 and 28 days, respectively, post-transplantation. No *sry* was detected in samples from sham and DMEM groups. However, relative to controls (DMSO pretreated group), a 2.7- and 1.9-fold increase in the copy number of *sry* was observed in the SRT1720 pretreated group at 1 and 3 days, respectively (Figures 2e and f). The *sry* was not detected in any samples at 28-day post-transplantation. These results indicate that pretreating aged hMSCs with SRT1720 promotes cell survival at early times after transplantation in the ischemic rat heart.

### Enhanced angiogenesis by SRT1720 pretreatment

**Figure 3:**
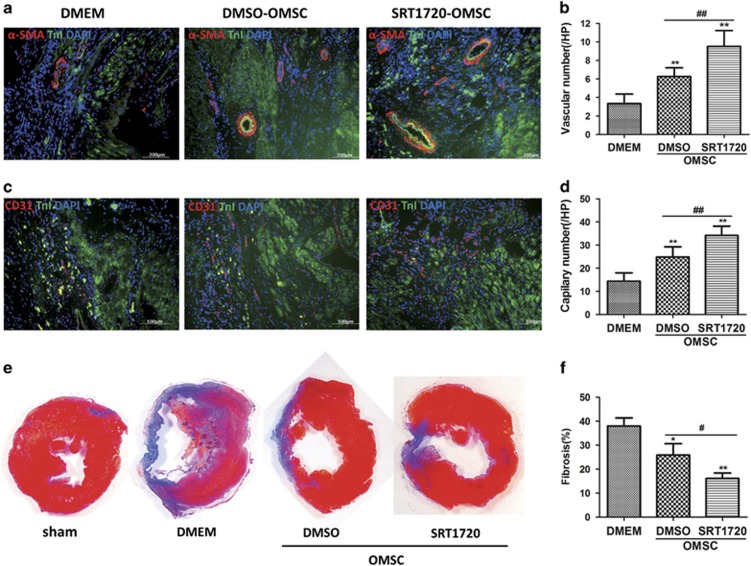
Improved angiogenesis and fibrosis of rat hearts after transplantation with SRT1720 pretreated aged hMSCs. Blood vessels in the border zone of ischemic hearts were stained with *α*-SMA (**a** and **b**) and CD31 (**c** and **d**). Cardiac remodeling was analyzed by Masson's trichrome staining (**e** and **f**). Data are expressed by mean±S.D. (*N*=6). *P<0.05 *versus* DMSO group; **P<0.01 *versus* DMSO group; ^#^*P*<0.05; ^##^*P*<0.01

Rat hearts were collected at 28 days after MI and cell therapy, and angiogenesis was assessed by quantifying blood vessel density around the infract zone using immunofluorescence staining of CD31 (a marker for vascular endothelial cells) and *α*-SMA (a marker for vascular smooth muscle cells) (Figures 3a and c). The density of CD31 and *α*-SMA staining were slightly increased in the DMSO-OMSC group and significantly increased in the SRT1720-OMSCs group (Figures 3b and d). These results indicate that pretreatment with SRT1720 enhanced the pro-angiogenic properties of aged hMSCs perhaps secondarily to improved survival and early engraftment.

### SRT1720 pretreatment of aged hMSCs confers reduced MI scar size

Myocardial tissue sections at 28 days following cell therapy were stained with Masson's trichrome staining to evaluate the interstitial fibrosis. As expected scar areas were larger in the DMEM treatment group compared to the sham group, but reduced in the hMSCs treated group (Figure 3e). When the collagen volumes were quantified, the scar size were reduced in the DMSO-OMSC group compared to DMEM group (DMSO-OMSC group 25.9±4.7% *versus* DMEM group 38±3.4%, *P*=0.014) and were reduced with greater significance in the SRT1720-OMSC group (SRT1720-OMSC group 16±2.3% *versus* DMEM group 38±3.4%, *P*=0.008) (Figure 3f). These results suggest that SRT1720 pretreatment of aged hMSCs more effectively alleviates cardiac fibrosis.

### SRT1720 pretreatment of aged hMSCs leads to improved cardiac function

**Figure 4:**
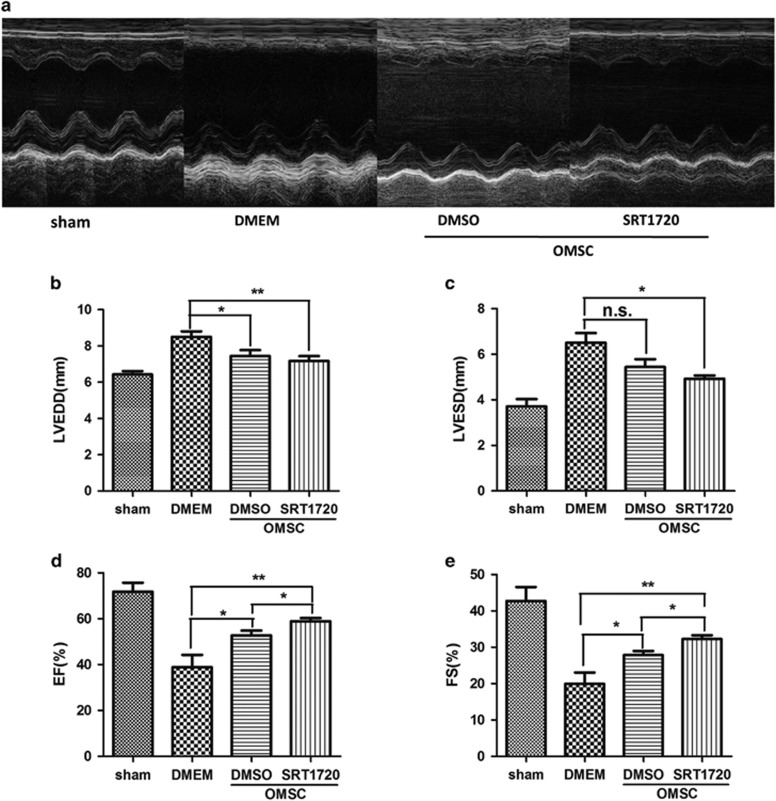
Enhanced cardiac function of rat hearts after SRT1720 pretreated aged hMSCs transplantation. Representative echocardiographic images are shown (**a**). The LVEDD (**b**), LVESD (**c**), LVEF (**d**) and LVFS (**e**) were analyzed, respectively. Data are expressed by mean±S.D. (*N*=6). *P<0.05; **P<0.01

Transthoracic M model echocardiography (Figure 4a) was conducted to evaluate cardiac dimensions (left ventricular end diastolic diameter (LVEDD) and left ventricular end systolic diameter (LVESD)) and functions (left ventricular ejection fraction (LVEF) and left ventricular fraction shorting (LVFS)) in the rat hearts following cell therapy. Compared to sham group, left ventricular dimensions were enlarged following MI (LVEDD from 6.43±0.44 mm to 8.11±0.87 mm, *P*<0.001; LVESD from 3.71±0.77 mm to 6.50±0.96 mm, *P*=0.007). Aged hMSC transplantation (DMSO pretreated) resulted in a trend for improvement of the ventricular dimensions compared to the DMEM group (LVEDD from 8.11±0.87 mm to 7.57±1.03 mm, *P*=0.014; LVESD from 6.50±0.96 mm to 5.44±0.84 mm, *P*=0.1) (Figures 4b and c). Notably, cardiac function of rats severely declined following MI compared to sham group (LVEF from 71.8±9.6% to 38.1±10.5%, *P*=0.002; LVFS from 42.7±9.4% to 20±7%, *P*=0.006), and only small improvements were observed following DMSO pretreated aged hMSC transplantation compared with the DMEM group (LVEF from 38.1±10.5% to 52.8±5%, *P*=0.028; LVFS from 20±7% to 27.9±2.8%, *P*=0.037; Figures 4d and e). However, when aged hMSCs pretreated with SRT1720 were transplanted into ischemic hearts, the cardiac dimensions were significantly decreased compared to the DMEM group (LVEDD from 8.11±0.87 mm to 7.17±0.64 mm, *P*=0.026; LVESD from 6.5±0.96 mm to 4.92±0.39 mm, *P*=0.019; Figures 4b and c) and, concomitantly, cardiac function significantly improved (LVEF from 38.1±10.5 to 58.9±3.6%, *P*=0.009; LVFS from 20±7 to 32.3±2.7%, *P*=0.018) compared to the DMEM group (Figures 4d and e).

### SRT1720 protects aged hMSCs against apoptosis

**Figure 5:**
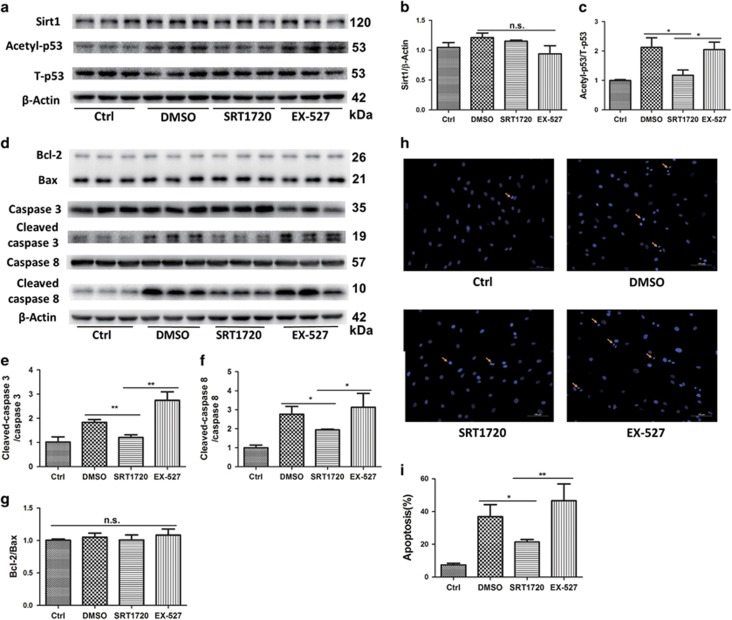
SRT1720 activates SIRT1 and protected aged hMSCs from apoptosis. SIRT1 expression (**b**) was detected by western blot (**a**), and SIRT1 activity was evaluated by the ratio of acety-p53/p53 (**c**) detected by western blot (**a**). Western blot (**d**) was carried out to quantify the apoptosis-associated protein cleaved caspase-3 (**e**), cleaved caspase-8 (**f**) involved in extrinsic apoptosis pathway and Bcl-2/Bax (**g**) involved in intrinsic apoptosis pathway. The apoptosis was also examined by Hoechst 33258 staining (**h** and **i**). Ctrl refers to normal culture medium. The other stress groups are treated with serum deprivation plus H_2_O_2_ medium. The volume fraction of DMSO was the same in every groups. Data are expressed by mean±S.D. (three independent experiments, *N*=3). *P<0.05; **P<0.01

To investigate the mechanism leading to improved cell survival following SRT1720 pretreatment of aged hMSCs, we first examined the expression and activity of SIRT1 under the stress condition of serum deprivation combined with 500 *μ*M H_2_O_2_. As shown in Figures 5a and b, no differences of SIRT1 expression were observed under the stresses when aged hMSCs were treated with or without SRT1720 and in the absence or presence of a SIRT1 specific inhibitor, EX-527, compared with DMSO pretreated group. P53 is a SIRT1 substrate that is closely linked to cell survival.^13^ Therefore, we evaluated acetylation of p53, as a surrogate marker of SIRT1 activity in aged hMSCs. The ratio of acetyl-p53(K382)/total-p53 was significantly decreased when cells were pretreated with SRT1720 and subsequently increased when pretreated with EX-527 (Figures 5a and c).

To expand upon our understanding of the role of SIRT1 in aged hMSCs, we assessed apoptosis following exposure to serum deprivation plus 500 *μ*M H_2_O_2_. The ratio of apoptotic cells detected by Hoechst staining in the SRT1720-OMSCs group was significantly lower than that observed in the DMSO-OMSCs group. Moreover, an increased ratio of apoptotic cells was observed upon pretreatment with EX-527 (Figures 5h and i). In keeping with these results, activation of caspase-3 and caspase-8 were also decreased following SRT1720 pretreatment and conversely enhanced following EX-527 pretreatment (Figures 5d–f). Notably, the Bcl-2/Bax ratio that is an indicator of intrinsic apoptosis was not changed (Figures 5d and g). These results suggest that SRT1720 promotes survival of aged hMSCs by inhibiting the extrinsic apoptosis and is dependent on SIRT1 activation.

### FAIM is necessary for the protective effects of SRT1720 on aged hMSCs

**Figure 6:**
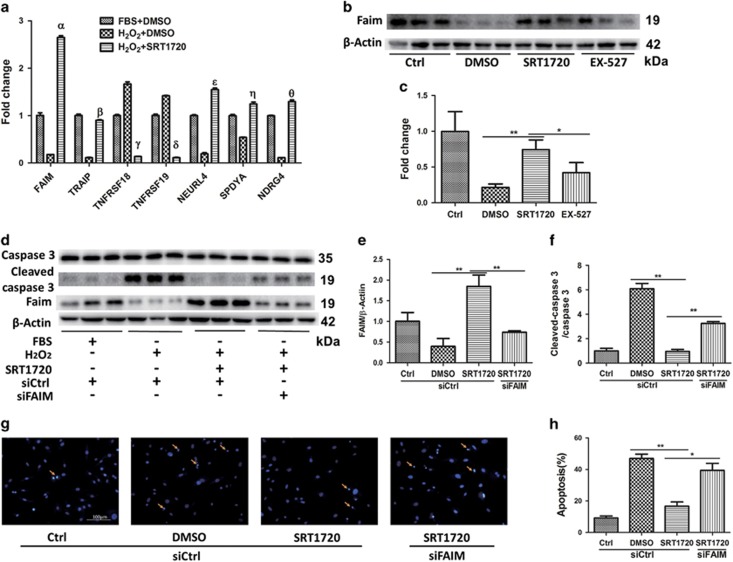
SRT1720 pretreatment protects aged hMSCs from apoptosis through upregulating FAIM. The expression of genes associated with cell survival and proliferation were confirmed by real time PCR (**a**). The expression of FAIM after SRT1720 pretreatment was tested by western blot (**b** and **c**). The reversal of the changed induced by SRT1720 was also evaluated when FAIM was silenced with western blot (**d**–**f**). Representative images of Hoechst 33258 staining (**g**) and quantitative analysis (**h**) are shown for apoptosis examination. Ctrl refers to normal culture medium. The other stress groups are treated with serum deprivation plus H_2_O_2_ medium. The concentration of DMSO (solvent of SRT1720 and EX-527) was the same in every groups. Greek letters refer to the fold change in H_2_O_2_+SRT1720 group *versus* H_2_O_2_+DMSO group, *α*=14.89±0.24; *β*=8.07±0.19; *γ*=12.34±0.31; *δ*=12.64±0.21; *ɛ*=8.05±0.25; *η*=2.31±0.22; *θ*=11.64±0.27. Data are expressed by mean±S.D. (three independent experiments, *N*=3). *P<0.05; **P<0.01

To further elucidate the mechanisms by which SRT1720 exerts its anti-apoptotic effect, we performed the transcriptome analysis of aged hMSCs pretreated with DMSO or SRT1720 for 24 h and then subjected to 0.5 mM H_2_O_2_ for 3 h. We identified 699 upregulated genes (>2-fold) and 645 downregulated genes (<0.5 fold) in the DMSO-H_2_O_2_ culture group (HD group) compared to DMSO with normal culture group (ND group) (Supplementary figure 3c). Compared to the HD group, there were 471 upregulated and 494 downregulated genes in the group of aged hMSCs with SRT1720 pretreatment plus H_2_O_2_ stress (HS group) (Supplementary figure 3c). Genes with dramatic changes in both HD *versus* ND groups and in HS *versus* HD groups are listed in the heat map (Supplementary Figure 3d). Among these, seven genes were associated with cell survival and apoptosis. We then validated these findings by quantitative real time PCR. Pro-apoptosis genes *TNFRSF19* and *TNFRSF18* were upregulated in serum deprivation with H_2_O_2_ culture group compared to normal culture and downregulated after SRT1720 pretreatment compared to vehicle control. Conversely, the anti-apoptosis gene *FAIM* and pro-survival or cell cycle genes *TRAIP*, *SPDYA* were downregulated in serum deprivation with H_2_O_2_ culture group compared to normal culture, and upregulated after SRT1720 pretreatment compared to vehicle control (Figure 6a). The anti-apoptosis gene *FAIM* demonstrated the most dramatic change in expression following SRT1720 pretreatment (14.9±0.2 folds *versus* DMSO pretreated group), and may account at least in part for the anti-apoptosis effects produced by SRT1720. Upregulation of FAIM was also observed at the protein level in aged hMSCs pretreated with SRT1720, while reduced protein expression of FAIM was observed in aged hMSCs pretreated with EX-527, as expected (Figures 6b and c).

To investigate whether FAIM mediated the anti-apoptosis effect of SRT1720 in aged hMSCs induced by H_2_O_2,_ a small interfering RNA targeted FAIM (siFAIM) was used. Compared to SRT1720 pretreatment group, the ratio of apoptosis cells in SRT1720 combined with siFAIM pretreatment group were significantly increased (Figures 6g and h) and activation of caspase-3 was also upregulated (Figures 6d–f). These results reveal that downregulation of FAIM mitigated the anti-apoptosis actions of SRT1720. Taken together, these results suggest that FAIM is necessary to mediate the protection of SRT1720 in aged hMSCs under oxidative stress.

## Discussion

Here we have shown that aged hMSCs have increased sensitivity to oxidative stress and this correlates with significantly decreased activity and expression of SIRT1. On the basis of these results and our previous reports,^15, 16^ we speculated that SIRT1 has an important role in the reduced stress resistance of aged hMSCs and the subsequent low survival of aged MSCs following cell transplantation *in vivo*. In support of this, we found that optimal treatment of aged hMSCs with the SIRT1 agonist SRT1720 enhanced cell survival in cultured cells under conditions of hypoxia and oxidative stress that mimic in part the conditions of ischemia and reperfusion that are present during MI *in vivo*. Enhanced survival *in vitro* was paralleled by enhanced cell therapy of aged hMSCs by SRT1720 pretreatment in a rat MI model of permament LAD ligation. H-MSCs pretreated with SRT1720 under optimal conditions conferred enhanced early survival and engraftment with lower scar and fibrosis and marked improvement of cardiac contractility at later time. We provide evidence that the enhanced functions conferred by SRT1720 involve enhanced activity but not expression of SIRT1 and improved protection against apoptosis perhaps mediated by enhanced expression of the FAIM by aged hMSCs induced by SRT1720 through SIRT1 in the aged cells. The results suggest a pharmacological approach to improve the potential of aged hMSCs for the treatment of cardiovascular disease including heart disease and stroke.

MSCs therapy for cardiovascular disease is through to be limited to a significant degree by the poor survival and engraftment of cells after transplantation, effects that are exacerbated by age.^5, 6^ In recent years, a variety of approached have been developed to improve such survival and engraftment of transplanted MSCs. Retroviral-mediated overexpression of the pro-survival gene Akt1 was first shown to enhance the survival of MSCs in an ischemic setting.^23^ Hypoxic pre-conditioning has also been used to improve the survival of multiple cell types after transplantation including MSCs.^24, 25^ Similarly, a variety of small molecules that enhance survival by blocking apoptosis have been shown to enhance MSCs engraftment and improve therapeutic efficacy.^26, 27^ To date and to our knowledge, no strategy has been described to specifically improve the survival of aged hMSCs in the setting of ischemic heart disease. Therefore, work described here is novel and significant.

Of all the synthesized SIRT1 activators, SRT1720 appears to be the most effective and shows promise in clinical application19. However, the actions of SRT1720 are cell-specific. SRT1720 has been shown to decrease the viability of multiple myeloma cells through caspase-3 mediated apoptosis^28^ and pancreatic cancer cells through a SIRT1-lysosomal-cell death pathway.^29^ SRT1720 appears to have a mainly protective effect in non-tumor cells. For example, SRT1720 ameliorated the senescence of airway epithelial cells,^30^ attenuated the endoplasmic reticulum stress and superoxide production of vascular endothelial cells,^31, 32^ and induced mitochondrial biogenesis in renal proximal tubule cells after oxidant injury.^33^ The latter results are consistent with our findings that SRT1720 protects aged hMSCs during subjection to ischemic/oxidative stress perhaps by suppressing the extrinsic apoptosis pathway and associated caspase-8 and its downstream caspase-3.

Our studies identify FAIM, an anti-apoptosis protein as the possible target for SIRT1 and mediator of cytoprotection by SRT1720. FAIM was first discovered in Fas-resistant B lymphocytes and is broadly expressed in multiple tissues.^34^ The sequence of FAIM gene is evolutionarily conserved from *C. elegans* to mammalian species, which supports a central role for the *FAIM* gene as a key apoptotic regulatory molecule.^34^ FAIM has an important role in altering the expression of c-FLIP and inhibiting fas-mediated apoptosis by binding to Fas to block its role in activating caspase-8 (in our studies, we found that the expression of apoptosis-associated cleaved caspase-3 and cleaved caspase-8 were decreased in SRT1720 pretreated aged hMSCs coincident with an upregulation of FAIM (Figures 5 and 6).^35^ A central role for FAIM was confirmed by our demonstration that siRNA-mediated downregulation of FAIM mitigated the anti-apoptosis effect of SRT1720 (Figure 6). These results confirm that FAIM is necessary for the protective effects of SRT1720.

### Conclusion and perspectives

We describe an effective pharmacological approach to pretreat aged hMSCs with SRT1720 that improves cell survival under oxidative stress and provides enhanced cell therapy. SRT1720 pretreated aged hMSCs demonstrated significantly improved survival and engraftment in ischemic hearts that lead to markedly preserved myocardial functions post MI. Our results suggest this modality of pretreatment has promising implications for clinical studies. It was recently demonstrated that SIRT1 and systemic SRT1720 conferred cardioprotection in a ischemia-reperfusion model.^36, 37, 38^ Therefore, it may even be feasible to combine pretreatment of MSCs with systemic administration of SRT1720 to provide an optimal therapy for MI. Our *in vivo* results are the first to describe safety and efficacy of SRT1720 pretreated aged MSCs in an MI model. The results support implementation of further mechanistic, pharmacological and toxicological studies in a large animal model prior to possible clinical application of this technology.

## Materials and Methods

### Animals and human bone marrow

Sprague–Dawley (SD) rats were purchased from the Zhejiang Academy of Medical sciences (Hangzhou, China). All experimental protocols were approved by the animal ethics committee of the second affiliated hospital of Zhejiang University in accordance with the Guide for the Care and Use of Laboratory Animals (NIH Publication No. 85-23, revised 1996). Human bone marrows were harvested from healthy donors or patients undergoing total hip replacement surgery (3 young healthy donors and 3 elder patients, as described in Supplementary Table 1). Informed consent was obtained from each donor and the protocol of processing human cells was approved by the ethics committee of the second affiliated hospital of Zhejiang University.

### Isolation and culture of MSCs

Bone marrows obtained from the proximal femur were washed with phosphate buffered saline (PBS), and mononuclear cells were separated using the bone marrow and cord blood nuclear cells isolation reagents (Wealthlin Science and Technologies, Toronto, Ontario, Canada) according to the user's guide. Then the collected MSCs enriched mononuclear cells were resuspended in low glucose Dulbecco's modified eagle medium (DMEM) (GIBCO, Life Technologies, Waltham, MA, USA) with 15% fetal bovine serum (FBS) (GIBCO, Life Technologies, USA). The cultures were plated on 10cm dish and maintained in a humidified atmosphere containing 5% CO_2_ at 37 ^o^C. After 24 h, non-adherent cells were removed and the remaining cells were cultured continuously. Cells were passaged at 70–80% confluence using 0.25% trypsin plus 0.2% EDTA and the 2–6 passage cells were used for experiments.

### Characterization of MSCs

H-MSCs at 6 passages were characterized for surface markers by a FACS Canto II Flow Cytometer (BD Bioscience, San Jose, CA, USA). Briefly, 1 × 10^6^ cells were collected and suspended in 100 *μ*l PBS with 1% FBS, and then stained with the surface molecular specific antibodies (mesenchymal surface markers: PE-CD29, PE-CD44, APC-CD90, endothelial cell surface marker: FITC-CD34, hematopoietic surface marker: FITC-CD45 and isotype-matched control) for 1 h. After twice wash with PBS, the expression of cell surface markers was quantified with FACSDiva software (BD Bioscience).

The differentiation of hMSCs into osteocytes, chondrocytes and adipocytes was conducted by the methods used in our previous study.^39^ Briefly, hMSCs were seeded in 6-well plate at density of 2 × 10^5^ per well, cultured until confluent. For the differentiation into adipocytes, hMSCs were treated with the adipogenesis induction medium for 23 days. The formation of fat vacuoles was visualized by oil red O staining. For osteogenesis differentiation, hMSCs were treated with osteogenesis induction medium for 3 weeks. Osteogenesis of hMSCs was evaluated with alizarin red S staining. Chondrogenesis differentiation of hMSCs was induced with chondrogenic induction medium for 3 weeks, and evaluated with toluidine blue staining. Images were captured with a light microscope.

### Establishment of cell stress models and cell survival assay

According to the commonly used models, three stress situations mimicking *in vivo* ischemic condition were developed *in vitro*. Aged hMSCs cultured in 96-well plate were exposed to normal culture or serum deprivation culture, serum deprivation culture under 0.1% O_2_ and serum deprivation culture with H_2_O_2_ for different time points. To maximize the effects of SRT1720, the appropriate conditions for stress were obtained when 50% cells survived under these stresses compared to normal culture. Cell survival was detected by cell counting kit 8 (CCK-8). Briefly, the medium was removed after stress and 100 *μ*l DMEM with 10 *μ*l CCK-8 (Dojindo, Minato-ku, Tokyo, Japan) solution were added to each well. After incubation for 2.5 h at 37 °C, the absorbance at 450 nm was measured by a microplate reader (Bio-Rad, Berkeley, CA, USA).

### Senescence-associated *β*-galactosidase staining

H-MSCs of the same passage (from younger and elder patients) were trypsinized and seeded into 6-well plate at 2 × 10^5^ cells per well. The next day, culture mediums were removed and cells were washed with PBS for three times and fixed with 4% formaldehyde for 15 min. After washing with PBS for three times, *β*-galactosidase staining was conducted with cell senescence-associated *β*-galactosidase staining kit (Byotime Biotechnology, Shanghai, China) per manufacturer's instructions. Cells were incubated at 37 °C overnight and washed with PBS for three times. Then imaging was performed with the inverted microscope (Leica, Wetzlar, Germany).

### SIRT1 activity assay

The nuclear protein was extracted from cells using Nuclear and Cytoplasmic Protein Extraction Kit (Beyotime Biotechnology, Shanghai, China) according to the manufacturer's protocols. The protein concentrations were determined by BCA method (Thermo Fisher Scientific, Waltham, MA, USA), and equal protein amounts from different samples were used to test the activity of SIRT1 according to the instruction of SIRT1 Fluorometric Drug Discovery Kit (Enzo Life Sciences, Farmingdale, NY, USA). Briefly, the nuclear protein solutions were incubated with standard acetylated substrate p53 protein (100 *μ*M) and co-substrate NAD^+^ (170 *μ*M) for 45 min at 37  °C, and then developer II was added to the system for another 45 min to terminate the reactions. The fluorescence intensity at 460nm excited by 360 nm light was measured using a fluorescence microplate reader (Molecular Devices, Sunnyvale, CA, USA).

### Pharmacological treatment of hMSCs

The SIRT1 specific activator SRT1720 and inhibitor EX-527 were all purchased from Selleck Chemicals (Houston, TX, USA), and dissolved into an organic solvent, dimethylsulfoxide (DMSO) before the treatment of hMSCs. To avoid the solvent disturbance of DMSO, concentrations of DMSO are less than 0.1% and the same in different treatment groups. Cells were exposed to fresh complete medium supplemented with different concentration of SRT1720 for different time points, followed by treatment with different cell stress models, to evaluate the optimal SRT1720 concentration and optimal timepoint by using CCK-8 test. For *in vivo* experiment, aged hMSCs were cultured in 10cm dishes before treatment. When cultured to 80% confluence, cells were pretreated with (SRT1720 group) or without SRT1720 (DMSO group), while the concentration of solvent DMSO is <0.1% and the same in both groups. After pretreated for 24 h, cells were collected in DMEM for implantation into rat hearts.

### Hoechst staining

For apoptosis detection, hMSCs were trypsinized and seeded into 24-well plate at 20 000 cells per well. After treatments, culture mediums were removed and cells were rinsed twice with PBS. Cells were fixed with 4% formaldehyde for 15 min followed by washing with PBS for three times. Overall, 100 *μ*l of 10 *μ*g/ml Hoechst 33258 (Life Technology, USA) was added and incubated at room temperature for 15 min. After three washes with PBS, the images were photographed with a florescence microscope (Leica, Wetzlar, Germany).

### RNA extraction and transcriptional profile analysis

Total RNA was isolated from hMSCs immediately after treatments using Trizol regent (Invitrogen, Carlsbad, CA, USA) according to the instructions. The concentrations and qualities of RNA were examined with NanoDrop 2000c spectrophotometer (Thermo Fisher Scientific, Waltham, MA, USA). Gene expression microarray was conducted with the Illumina Whole-Genome Gene Expression Direct Hybridization Assay system (Illumina, SanDiego, CA, USA). In brief, 500 ng of total RNA were reverse transcribed and synthesized to double-stranded complementary DNA (cDNA). After purification, cDNA were used to amplify and label multiple copies of biotinylated cRNA by *in vitro* transcription. After quantification, the labeled cRNA were dispensed to BeadChips and incubated for 14–20 h at 58 °C. After washing, the BeadChip was bind by Cy3-SA and the signal was detected by the iScan System and BeadArray Reader (Illumina, SanDiego, CA, USA). The gene expression data from scanned microarray was analyzed using Illumina's Genome Studio Gene Expression Module. The raw data were normalized using the quantile method to minimize the effects of variation arising from non-biological factors. Differential expression algorithms were used to compare a group of samples (referred to as the condition group) to a reference group, and the comparison was done using the error model of detection *P*-value. Differential expression was defined by fold change between groups which was more than 2 or <0.5. Clustering analysis was also conducted to examine the similarity of differential expression genes (Supplementary Figures 2A and B).

### Small interfering RNA transfection

Small interfering RNA (siRNA) was applied to silencing the expression of FAIM, and lipofectamine 2000 regent (Life Technology, Waltham, MA, USA) was used to transfect the FAIM specific siRNA (5′-GGGUGAGUUUGUAGAUGAUTT-3′) and negative control siRNA (5′-UUCUCCGAACGUGUCACGUTT-3′) into hMSCc according to the manufacturer's instructions. The optimized concentration of FAIM specific siRNA for transfection was determined by real time PCR and western blot. In brief, 1 day prior to siRNA transfection, hMSCs were plated into 6-well plates in normal medium to attain 50–60% confluence when transfecting the next day. Then, 50 pmol of siRNA with 250 *μ*l Opti-MEM (Life Technologies) and 2.5 *μ*l of lipofectamine 2000 (Life Technologies) with 250 *μ*l Opti-MEM were mixed for 20 min at room temperature and added to each well. After incubation for 5 h, the complexes were removed and fresh medium with 15% FBS were added. Overall, 24 h after transfection, total RNA were extracted from hMSCs and tested by real time PCR. Total protein was isolated and detected by western blot 48 h after transfection.

### Quantitative real time RT-PCR

Prior to PCR, 1 *μ*g of RNA was reverse transcribed to cDNA using the PrimeScript RT reagent Kit (Perfect Real Time, Takara, Japan) per manufacturer's instructions. cDNA was diluted by 5-folds and used for quantitative real time PCR using the SYBR Premix Ex Taq (2 ×) reaction mix (Takara, Japan) on the 7500 Fast real time PCR system (Applied Biosystems, Carlsbad, CA, USA). The reaction conditions included: 95 °C for 10 min and then 40 cycles of 95 °C for 15 s, 65 °C for 30 s and 72 °C for 10 s. Primers were designed with the Primer 3 Input (version 4.0.0) online software (http://primer3.ut.ee/) and the sequences were listed in Supplementary Table 2. The expression of target genes was determined by the comparative ΔΔCt method and *β*-ACTIN was used as the control gene.

### Western blotting analysis

Cells were homogenized in ice-cold lysis buffer supplemented with proteinase inhibitor. After 30 min' incubation, cell lysates were centrifuged at 12 000 *g* for 30 min at 4 ^o^C to remove debris. Protein concentrations were measured by BCA method (Thermo Fisher Scientific), separated by sodium dodecyl sulfate polyacrylamide gel electrophoresis (SDS-PAGE) with a current of 30 mA and transferred onto polyvinylidene difluoride (PVDF) membranes (Merck Millipore, Darmstadt, Germany) electrophoretically with a current of 300 mA. After blocking with 5% bovine serum albumin (BSA) for 1 h, the membranes were incubated with primary antibodies at a concentration according to the product instructions overnight at 4 °C. The membranes were washed three times with PBS-Tween (PBST) and incubated with corresponding HRP-conjugated secondary antibodies (1:5000) for 1 h at room temperature. After being washed three times with PBST, the protein bands were detected using the Gel Doc EZ Imaging System (Bio-Rad, Berkeley, CA, USA) with an ECL kit (Merck Millipore, Germany) and analyzed using Image Lab software (Bio-Rad).

Mouse monoclonal anti-SIRT1 antibody, Mouse monoclonal anti-cleaved-caspase-8 and rabbit monoclonal anti-acetyl-p53 (K382) antibody were obtained from Abcam (Cambridge, UK). Rabbit monoclonal anti-Bcl-2 antibody, rabbit monoclonal anti-Bax antibody, rabbit monoclonal anti-cleaved-caspase-3 antibody and rabbit monoclonal anti-FAIM antibody were obtained from Cell Signaling Technology (Beverly, MA, USA). Mouse monoclonal *β*-actin antibody was obtained from BD Biosciences (San Jose, CA, USA), horseradish peroxidase (HRP)-conjugated anti-mouse and anti-rabbit secondary antibodies were obtained from Cell Signaling Technology.

### MI model and hMSCs transplantation

Rats MI model were established by ligation of the left anterior descending (LAD) coronary artery of female SD rats (8 weeks old, 220–250 g) as previously described.^40^ All 60 female SD rats were randomly divided into four groups: the sham group (sham) received the same thoracotomy surgery without ligaturing the LAD coronary or any other intervention (*n*=15); the DMEM group (DMEM) received coronary ligation and DMEM injection (*n*=15); the DMSO pretreated aged MSCs group (DMSO-OMSCs) received coronary ligation and injection of male aged hMSCs pretreated with DMSO (*n*=15); the SRT1720 pretreated aged MSCs group (SRT1720-OMSCs) received coronary ligation and injection of male aged hMSCs pretreated with SRT1720 (*n*=15). Rats MI model were established by ligation of the LAD descending coronary artery of female SD rats (8 weeks old, 220–250 g). In brief, the hearts of rats were exposed by limited thoracotomy with tracheal intubation under general anesthesia with pentobarbital (50 mg/kg), and the LAD coronary artery was ligatured with a 6-0 silk suture. After 30 min, the ischemic border zone of hearts were intramyocardially injected with 150 *μ*l DMEM or 10 million aged male hMSCs using a 30-gauge needle at five sites. After the surgery, the chest was closed and a warming pad was arranged for the recovery of rats.

### Measurement of SRY gene by quantitative real time PCR

Human sex determination SRY gene copy number in female rat heart tissues following hMSCs transplantation was measured by real time PCR. In brief, the whole heart tissues from different groups were frozen and pulverized with liquid nitrogen. Genomic DNA was extracted using MiniBEST Universal Genomic DNA Extraction Kit Ver.5.0 (Takara, Japan) according to the manufacturer's instructions. Real time PCR was performed with SYBR green reaction mix using 7500 fast real time PCR system (Applied Biosystems, Carlsbad, CA, USA) according to manufacturer's instructions. Primers used include: human SRY gene forward primer 5′ GGTAAGTGGCCTAGCTGGTG 3′, reverse primer 5′GATCCCGCTTCGGTACTCTG 3′ rat 36B4 gene: forward primer 5′-CTCACTCCATCATCAATGGATACAA-3′, reverse primer 5′-CAGCCAGTGGGAAGGTGTAGTCA-3′. The reaction conditions were 95 °C for 30 s followed by 40 cycles of 95°C for 5 s, 56°C for 31 s and 95°C for 15 s. Engrafted hMSCs numbers were evaluated using the comparative ΔΔCt comparative method.

### Echocardiographic examination

Cardiac function of rats at the 28th day following cell therapy were evaluated by transthoracic two dimensional echocardiography (Vevo 2100; VisualSonics, Toronto, Ontario, Canada) under general anesthesia with 2% isoflurane in O_2_ gas. The measurement of LVEDD, LVESD were conducted in at least three cycles. LVESV and LVEDV were calculated according to the M-mode formula of Teichholtz: 7 × LVEDD^3^/(2.4+LVEDD) and 7 × LVESD^3^/(2.4+LVESD), respectively.^41^ LVEF and LVFS were calculated by ((LVEDD−LVESD)/LVEDD) × 100 and ((LVEDV−LVESV)/LVEDV) × 100, respectively. The echocardiographic analysis was performed in a double-blinded manner. The animal surgery, echocardiographic examination, and data analysis were conducted by three different researchers, respectively.

### Masson's trichrome staining

Scar size of the rat hearts were evaluated by Masson's Trichrome staining as previously described.^42^ In brief, hearts were collected from rats of different groups and made into frozen sections of 7 *μ*m. Then Masson's Trichrome staining was performed using Masson Trichrome Kit (Sigma Aldrich, St. Louis, MO, USA) according to the manufacturer's instructions. The percentage of fibrosis in the whole left ventricular was quantified with ImagePro-Plus software (Media Cybernetics, Rockville, MD, USA).

### Immunofluorescence staining

Frozen sections from rat hearts were fixed with 4% formaldehyde for 15 min and permeabilized with 0.25% Triton X100 for 20 min. The slices were washed with 0.05% PBST for three times and blocked with 5% BSA in PBST for 1 h. The sections were incubated with specific primary antibody (1:100) overnight at 4°C. After three washes in PBST, the sections were incubated with the corresponding secondary antibody for 1 hour at room temperature. After three washes, the nucleus was stained with 4′,6-diamidino-2-phenylindole (DAPI). Fluorescent imaging was performed using a fluorescent microscope (Leica, Wetzlar, Germany). The primary and secondary antibodies used in this experiment were obtained from Abcam (Cambridge, UK).

### Statistical analysis

All data are shown as mean±S.D. and analysis was implemented with Graphpad Prism 5 software (GraphPad Software, La Jolla, CA, USA). A normal distribution was confirmed by the Shapiro–Wilk test for each set of the obtained data from each studied group. Student's *t-*test was used to compare between two groups, and one-way analysis of variance (ANOVA) was performed to analyze the difference among more than two groups followed by multiple comparisons using the Student–Newman–Keuls test. Differences were considered significant when *P*<0.05.
