# Supplementary material for: SRT1720 promotes survival of aged human mesenchymal stem cells via FAIM: a pharmacological strategy to improve stem cell-based therapy for rat myocardial infarction
Source: Cell Death Dis. 2017 Apr 6;8(4):e2731–. doi: 10.1038/cddis.2017.107 (PMC5477573; doi:10.1038/cddis.2017.107)
Supplement: Supplementary Information [file cddis2017107x1.docx]

**Supplementary table 1 Characteristics of patients for MSCs isolation**

| Donors Age (years) Gender Diseases Group |
| --- |

Y_1_ 23 female Healthy YMSC

Y_2_ 26 male Healthy YMSC

Y_3_ 25 male Healthy YMSC

O_1_ 81 female Femoral neck fracture OMSC

O_2_ 75 male Femoral head necrosis OMSC

O_3_ 73 male Osteoarthritis OMSC

|  |
| --- |
