# Supplementary material for: SRT1720 promotes survival of aged human mesenchymal stem cells via FAIM: a pharmacological strategy to improve stem cell-based therapy for rat myocardial infarction
Source: Cell Death Dis. 2017 Apr 6;8(4):e2731–. doi: 10.1038/cddis.2017.107 (PMC5477573; doi:10.1038/cddis.2017.107)
Supplement: Supplementary Table 1 [file cddis2017107x2.docx]

**Supplementary table 2 Primer sequences for real time RT-PCR**

| Gene Product  name Gene ID Primer sequence (5’-3’) size (bp) |
| --- |
| FAIM NM_001033030.1 Forward AGCTGCAAAGACAAAAGCGA 232  Reverse TACAAACTCACCCGCTGTCT  TRAIP NM_005879.2 Forward CTAAAAGAGGCACGGAAGGC 199  Reverse TTTCCTGCAGCATCGTTAGC  TNFRSF19 NM_018647.2 Forward GTCAACCTCGTGAAGATCGC 242  Reverse GCTCTGTGGGCATATTCGTG  TNFRSF18 NM_148901.1 Forward TGTCCAGCCTGAATTCCACT 176  Reverse CTGTCCAAGGTTTGCAGTGG  NEURL4 NM_032442.2 Forward CAACGGTCTCAAGATCTGCG 215  Reverse GCTCAGGGTTCACGATTGTC  NDRG4 NM_020465.2 Forward AACTTCGAGGACATGCAGGA 224  Reverse ATGAGTGCAAACTTGGCCAG  SPDYA NM_001008779.1 Forward AGCCCATTACTCTGAAGCGT 193  Reverse TTACAGCAGCAGTCCATCCA |

β-ACTIN [NM_001101.3](http://www.ncbi.nlm.nih.gov/entrez/viewer.fcgi?db=nucleotide&id=168480144) Forward CCCTGGAGAAGAGCTACGAG 180

Reverse CGTACAGGTCTTTGCGGATG

|  |
| --- |
