# Supplementary material for: SRT1720 promotes survival of aged human mesenchymal stem cells via FAIM: a pharmacological strategy to improve stem cell-based therapy for rat myocardial infarction
Source: Cell Death Dis. 2017 Apr 6;8(4):e2731–. doi: 10.1038/cddis.2017.107 (PMC5477573; doi:10.1038/cddis.2017.107)
Supplement: Supplementary Table 2 [file cddis2017107x3.docx]

SUPPLEMENTARY FIGURE LEGENDS

**Supplementary figure 1** The differences between aged (OMSC) and young MSCs (YMSC). Senescence of hMSCs was analyzed by SA-β-galactosidase staining (a, b). The viability of hMSCs under serum deprivation combined with 500 μM H_2_O_2_ for 3 hours was evaluated by CCK-8 (c). SIRT1 activity of hMSCs was examined with the SIRT1 Fluorometric Drug Discovery Kit (d). Western blot was conducted to detect the expression of SIRT1 from young and aged hMSCs (e). Data are expressed by mean ± SD (Three independent experiments, N=3). *, p<0.05 vs. young hMSCs; **, p<0.01 vs. young hMSCs.

**Supplementary figure 2** The survival of aged hMSCs under different conditions. Cell viabilities were detected by CCK-8. Survivals of aged hMSCs under conditions of different time of serum deprivation (a), serum deprivation with hypoxia (b), serum deprivation with different concentration of H_2_O_2_ (c) are shown. Data are expressed as mean ± SD (Three independent experiments, N=3). *, p<0.05; **, p<0.01.

**Supplementary figure 3** The transcriptional profile of aged hMSCs after SRT1720 pretreatment. The heatmaps of the differential expression genes in ND group versus HD group (a) and HD group versus HS group (b) are shown. The ups and downs of genes involved in the transcriptional profile of aged hMSCs with or without pretreatment are shown by Venn diagram (c). Genes with more than 3 folds change regulated both by DMSO or SRT1720 were presented in the heatmap (d). ND refer to DMSO with normal medium group; HD refer to DMSO with H_2_O_2_ group; HS refer to SRT1720 with H_2_O_2_ group.
