# Supplementary figures and images for: SRT1720 promotes survival of aged human mesenchymal stem cells via FAIM: a pharmacological strategy to improve stem cell-based therapy for rat myocardial infarction
Source: Cell Death Dis. 2017 Apr 6;8(4):e2731–. doi: 10.1038/cddis.2017.107 (PMC5477573; doi:10.1038/cddis.2017.107)

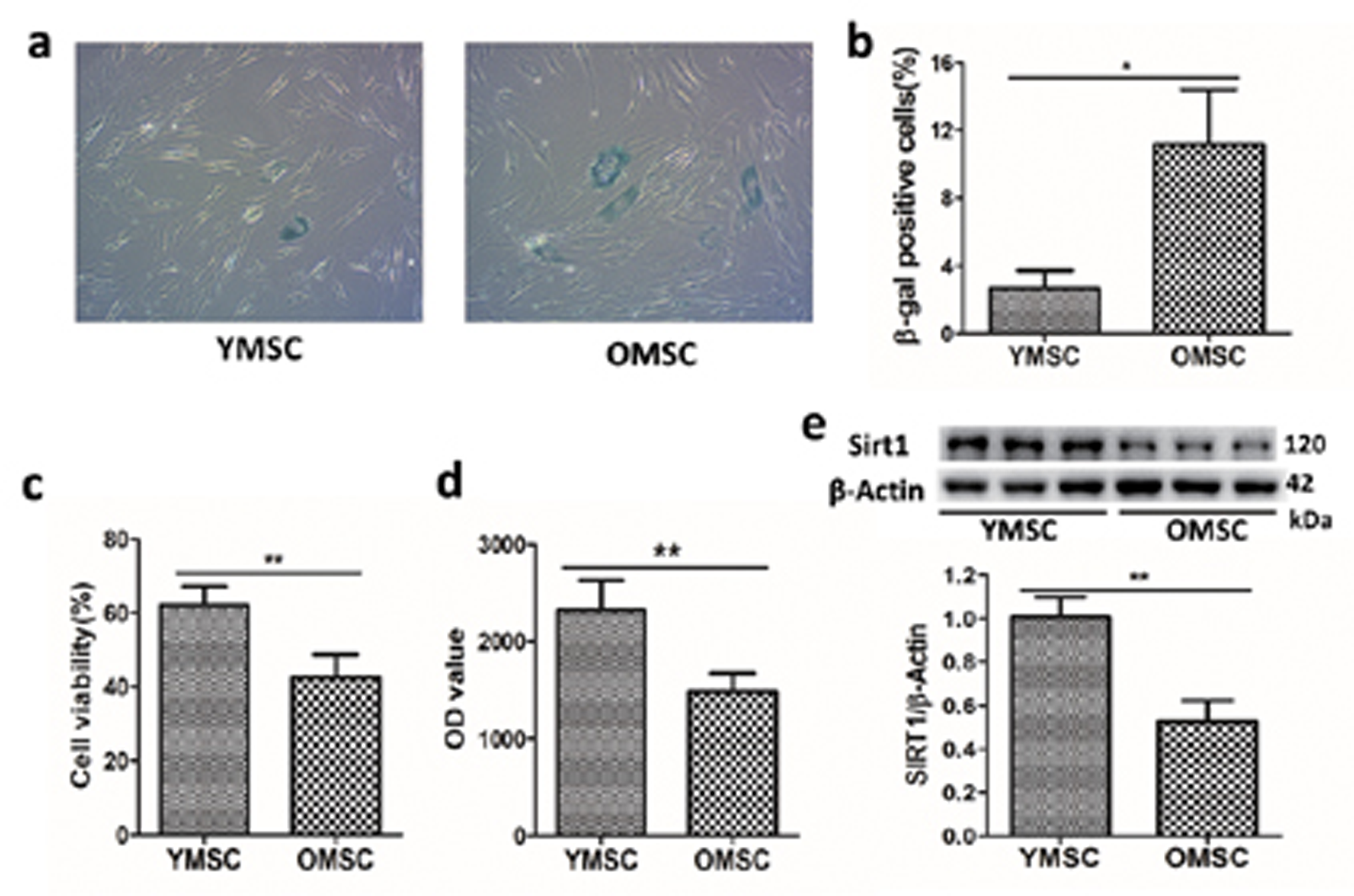

Supplement: Supplementary Figure 1 [file cddis2017107x4.tif]

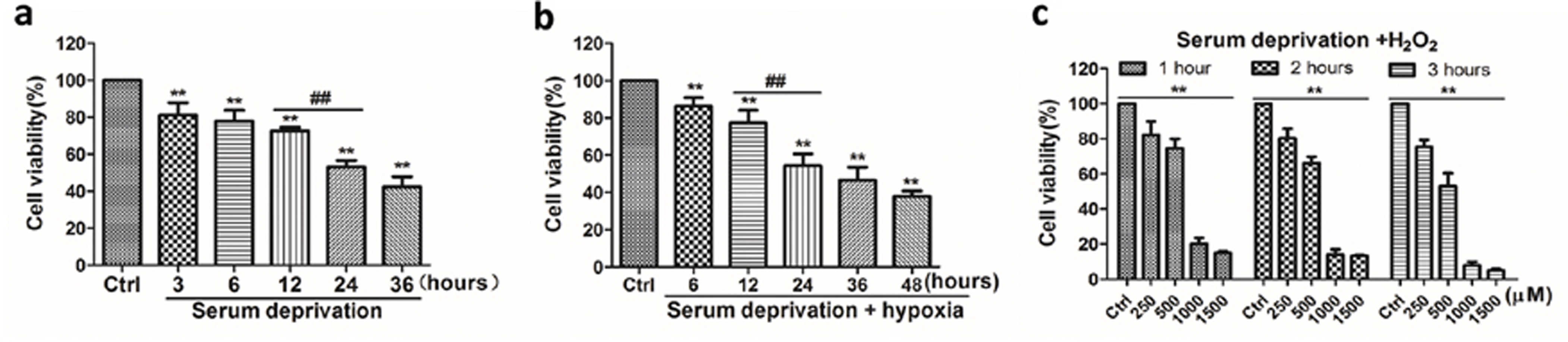

Supplement: Supplementary Figure 2 [file cddis2017107x5.tif]

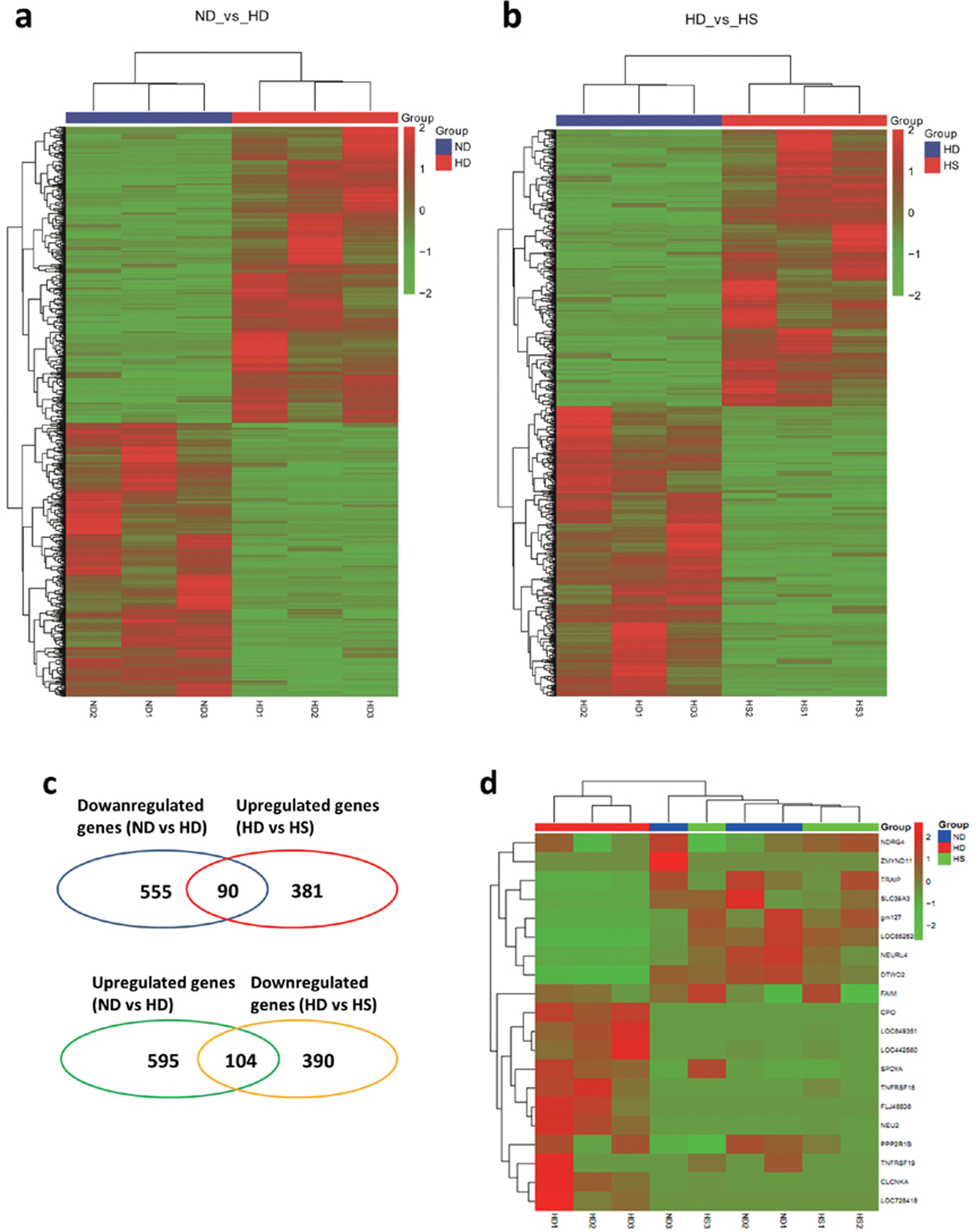

Supplement: Supplementary Figure 3 [file cddis2017107x6.tif]
